# Occupational Burnout Syndrome in Polish Physicians: A Systematic Review

**DOI:** 10.3390/ijerph16245026

**Published:** 2019-12-10

**Authors:** Magdalena Zgliczyńska, Stanisław Zgliczyński, Michał Ciebiera, Katarzyna Kosińska-Kaczyńska

**Affiliations:** 1 Second Department of Obstetrics and Gynecology, The Center of Postgraduate Medical Education, 01-809 Warsaw, Poland; zgliczynska.magda@gmail.com (M.Z.); kasiakosinska@wp.pl (K.K.-K.); 2 Chair and Department of Experimental and Clinical Physiology, Laboratory of Center for Preclinical Research Medical University of Warsaw, 02-097 Warsaw, Poland; 3 Department of Internal Diseases and Endocrinology, Central Teaching Clinical Hospital, Medical University of Warsaw, 02-097 Warsaw, Poland; stanislaw.zgliczynski@gmail.com

**Keywords:** burnout, occupational burnout, depersonalization, emotional exhaustion, personal accomplishment, physicians, Poland

## Abstract

Due to the nature of their work, physicians are exposed to chronic stress. This may potentially lead to the widespread occurrence of occupational burnout syndrome (BS). The aim of this systematic review study was to summarize available published data concerning the prevalence of BS in Polish doctors. The literature search was performed using the following databases: PubMed/MEDLINE, Scopus, Cochrane Central Register of Controlled Trials (CENTRAL) and Google Scholar. The last search was performed on September 27th, 2019. Only articles in English or Polish on graduated doctors practicing in Poland were taken into account. All types of original research were considered eligible. However, review articles, book chapters, case reports, case series, conference papers, study protocols and articles in languages other than English and Polish were excluded. There were no restrictions on age, seniority or specialty of study participants. The literature search revealed a total of 21 studies that met the inclusion criteria. The results of individual studies were very diverse, which makes it difficult to draw specific conclusions. However, the problem of burnout among Polish doctors is valid and worth special attention from society, health policy leaders, and doctors themselves. High-quality research is essential to for a better understanding of this topic.

## 1. Introduction

The phenomenon of occupational burnout syndrome (BS) was first described in 1974 by Freudenberger [1], as a state of mental and physical exhaustion caused by one’s professional life and exposure to prolonged stress. It was a trigger for further attempts to define this matter. Therefore, due to the complex nature of the condition, various concepts and definitions of BS have been developed to date and, consequently, also multiple diagnostic tools have been implemented. According to one of the most popular and widely recognized theories created by Maslach (1976), BS is a three-dimensional set of symptoms that occurs fairly regularly in people employed in occupations involving work in human services. It is characterized by emotional exhaustion (EE), depersonalization (DP) and a sense of low personal accomplishment (PA) [2]. The Maslach Burnout Inventory (MBI) for Human Services Survey is currently the most widely used tool for research in this field [3]. It allows the determination of the severity of the three above-mentioned BS symptoms. Gil Monte described a different perspective on BS. He created the Spanish Burnout Inventory (SBI) [4] and distinguished four areas of BS: enthusiasm toward the job, emotional exhaustion, indolence and guilt. The Italian Link Burnout Questionnaire (LBQ) by Santinello also considers four burnout aspects, which are distinct from SBI: psychophysical exhaustion, deterioration of relationships, sense of professional failure and disillusion [5]. On the other hand, Steuden and Okła distinguished five BS manifestations: reduced emotional control, loss of subject’s commitment, decreased effectiveness, limited interpersonal contacts and physical fatigue [6]. For the purpose of the unification of this review, the authors decided to focus primarily on the concepts used in the data included.

Due to the nature of their work, physicians are a professional group that is highly exposed to BS. According to a recently published systematic review, the overall BS prevalence in physicians ranged from 0% up to even 80.5% [7]. The review included only one study on the population of Polish doctors by Glebocka [8]. Polish physicians might to be particularly vulnerable to BS due to the poor condition of the healthcare system in Poland. According to the latest Euro Health Consumer Index 2018, which is an attempt at measuring the performance of the healthcare system, the Polish healthcare system was ranked 32nd out of the 35 countries included in the report [9]. In 2016, Poland was one of the European Union (EU) countries with the lowest health expenditure per capita, with only Latvia, Romania, Bulgaria and Croatia ranking lower [10,11]. Moreover, numbers of practicing doctors are lower compared to the majority of neighboring countries and, admittedly, insufficient. In 2016, Poland had one of the lowest ratio of practicing physicians per 1000 people among the EU countries—2.4 compared to the average of 3.6 in the EU [12,13]. This contributed to repeated strikes by Polish doctors in the past few years in which the following postulates were presented: Increased expenditures on healthcare; achievement of average indicators of the length and quality of life at least at the level of the European Union; higher standards of care and prevention; solving the problem of shortages in medical staff; and improvement in working conditions and wages [14,15,16,17].

Delving into the essence of this problem seemed particularly important to the authors as physicians’ well-being is considered as a forgotten quality factor in healthcare [18]. It was proved that BS may lead to reduced quality of life in physicians [19,20], the occurrence of depression symptoms [21] or even suicidal behaviors [22]. Even the potential overlapping of depression and burnout is also being widely discussed [21,23]. There is evidence that it negatively correlates with the feeling of empathy for patients [24]. Moreover, it may influence patient satisfaction and interpersonal aspects of care [25,26]. Furthermore, it can be associated with reduced work efficiency [27,28], an increased frequency of medical errors [29,30,31,32] and worse patient outcomes [33,34]. 

The aim of this systematic review study was to summarize available data concerning the occurrence of BS in the population of physicians working in Poland.

## 2. Materials and Methods 

The systematic literature review of research articles concerning the phenomenon of BS in Polish physicians was performed with the use of the following databases: PubMed/MEDLINE, Scopus, Cochrane Central Register of Controlled Trials (CENTRAL) and Google Scholar. The last search was performed on September 27th, 2019. It was not necessary to contact the authors of selected works to obtain additional information. This review was written in accordance with the Preferred Reporting Items for Systematic Reviews and Meta-Analyses (PRISMA) statement.

The search strategy was tailored to the specific database (details provided in Table 1).

All types of original research articles were considered eligible. Review articles, book chapters, case reports, case series, conference papers and study protocols were excluded from further considerations. Only articles written in English or Polish were taken into account. No publication date restrictions were imposed. Only studies on graduated doctors practicing in Poland were taken into account, while research articles on mixed groups of healthcare professionals, other medical professions and students of medicine and other faculties related to health care were excluded from further analyses. There were no restrictions on the age, length of service or specialty of study participants.

Titles and abstracts of research papers obtained as a result of the search described above were screened independently by two study authors. Full-text versions of studies that appeared to be potentially eligible based on the first stage of evaluation were individually assessed by three other authors. For each study, two authors collected the baseline characteristics of participants and outcomes. The developed data extraction sheet was pilot-tested on five randomly-selected included studies and refined accordingly. Extracted information included: study population and comparator demographics, recruitment, methodology, diagnostic tools used and outcomes.

Two authors individually assessed the risk of bias in selected studies using a modified version of the Newcastle-Ottawa Scale [35] based on a large systematic review on a similar topic—"Prevalence of Burnout Among Physicians. A Systematic Review” by Rotenstein et al. [7]—additionally adapted by the authors for the needs of this systematic review. Any disagreements were resolved through discussion and consensus with all study authors. 

## 3. Results

### 3.1. Database Search Results

The designed systematic literature search identified a total of 21 studies that met all the inclusion criteria. Selected studies included a total of 1674 doctors practicing in Poland, of at least 14 different specialties. Two series of publications referred to the same study group: the first one consisted of 2 publications, the second one consisted of 5 publications. The search of PubMed/MEDLINE, Scopus, Cochrane Central Register of Controlled Trials (CENTRAL) and Google Scholar provided a total of 329 citations. After adjusting for duplicates (using an automatic EndNote X9 duplicate search followed by manual verification), 220 remained, of which 66 full-text articles were assessed for eligibility. Other details regarding the selection process are included in the adapted PRISMA flow chart presented in Figure S1.

### 3.2. General Characteristics of the Included Studies

A summary of the characteristics of research articles included in the review is provided in Table 2.

### 3.3. Summary of Individual Study Results

The results of studies that considered the prevalence and severity of BS are summarized in the table below (Table 3).

### 3.4. Risk and Protective Factors against BS

The vast majority of articles determined risk as well as protective factors for the occurrence of BS. Details are provided in Table 4.

### 3.5. Risk of Bias Assessment

The risk of bias assessment was performed using a modified version of the Newcastle-Ottawa Scale [35] based on Rotenstein et al. [7] and adapted by the authors for this review. A detailed bias assessment is available in Table S1 and Table S2. Nevertheless, a vast majority of the studies were of medium or low credibility. We evaluated five aspects: representativeness, sample size, non-respondent description, tools used for the detection of BS and statistics. Possible scores in each category were 0 or 1 point. Only one study obtained the maximum number of points (5/5). Two series of articles (two and five articles each) and one separate article received 4/5 points. Three and two points were scored by five and seven studies, respectively. All studies were cross-sectional, and therefore constituted a III Level of Evidence, which indicates a B/C Grade of Recommendation.

## 4. Discussion

The scientific papers included in this review differ primarily in their methodology, as well as in results. Six different tools were used to examine the level of BS in Polish physicians and, in addition, the most popular tool—MBI—also appeared in a few different versions. The following paragraphs are an attempt to interpret the collected results with a division into sub-sections.

### 4.1. BS in Physicians in Poland and Worldwide

As it was mentioned, at present Polish health care is facing many problems. That is why we considered it important to determine whether Polish doctors are therefore particularly vulnerable to burnout. There is a lot of evidence that exposure to BS varies depending on the country [56,57,58]. In a large meta-analysis by Rotenstein et al., overall BS stratified by country ranged from 4% in Peru up to 61% in Canada. Similarly, large differences could be found in classic BS components (EE: 14%—Peru and 72%—Palestine; DP: 9%—Greece, 35%—Poland and 79%—Turkey; sense of low PA: 8%—Australia and 77%—India) [7]. In our review few papers reported the overall BS occurrence. In a study by Makara-Studzinska et al. every second physician showed a high level of BS [55], while Czekajska-Chehab et al. reported 37% of the examined population [36]. On the other hand, Leszczynski et al. reported 16% [54], whereas Misiolek et al. reported 18% [52]. Nevertheless, a vast majority of the articles focused on the severity of individual symptoms. A high level of EE in Polish doctors in research articles included in this review ranged from 22% [40] to 52% [42], whereas a high level of DP oscillated between 34% [41] and 62% [40]. A large dispersion could result from study population variety and different diagnostic tools and cut-off points used as presented in Table 3. Only one study containing a direct comparison of Polish physicians to physicians from other countries was identified. In a study by Soler et al., Polish general practitioners and family doctors were scored as the 4th highest in terms of the severity of EE, 8th in terms of DP and 5th in terms of PA out of doctors from 12 European countries [41]. Nevertheless, more high-quality comparisons are necessary to reach any consensus on this matter. 

### 4.2. Gender and BS in Physicians

A meta-analysis published by Purvanova et al. on the relationship between gender and BS in various other professions revealed slightly higher EE in women; however, at the same time, a lower level of DP [59]. However, the situation in the medical environment is not entirely clear. The recent Medscape National Physician Burnout, Depression and Suicide Report (2019) on over 15,000 doctors showed that burnout was self-reported by 50% of women and 38% of men physicians. In the scientific literature, studies reporting either no significant differences between the sexes [60,61] as well as indications that female doctors are more sensitive to burnout [62,63,64] are also available. Among the studies included in this review results also varied. Misiolek et al. did not find significant differences between male and female doctors in any variable [51,52] while the international study by Soler et al., which also included Polish doctors, pointed to the male sex as being a BS risk factor [41]. Conversely, studies suggesting that female doctors may suffer from a higher severity of EE [37] and lower satisfaction from PA [37,42] in comparison to male doctors were also retrieved. Such divergent results obtained on different populations may result, for example, from the medical specialty of the studied group or cultural background. Nevertheless, the Supreme Medical Council of Poland report of 10 September 2019 shows that up to 58% of doctors practicing in Poland are women [65]. Despite the lack of a final conclusion in this matter, it may be worth looking more closely for BS symptoms in this group, particularly in the face of growing staff shortages in Poland.

### 4.3. Specialty and BS in Physicians

The retrieved studies suggested a higher risk of BS in anesthesiologists [43] and neurologists [39], which was also reflected in studies from other countries [66,67]. However, it should be noted that articles selected for this systematic review did not cover all medical specialties. Nevertheless, in a study conducted by Walocha et al. [44], the highest overall level of burnout was found in non-surgical specialists. Worldwide, research has indicated some medical specialties associated with a particular burden, which are, among others: front line of healthcare (family medicine, internal medicine, emergency medicine) [68], cancer professionals [69], psychiatry [70] and surgery [71]. 

Currently, Polish doctors can undertake postgraduate education in 77 different specializations. Our survey covered at least 14, though we still lack a full view. Except specialization itself, the setting of the medical practice may also play a significant role [71,72]. Currently, many Polish doctors tend to work only in the private sector, with greatly different work characteristics than in the public sector, and such comparisons have not been yet thoroughly investigated. 

### 4.4. Professional Experience and BS in Physicians

In a study by Wojtyna et al. [42] no differences were found between doctors during specialty training and specialists. Conversely, Mroz et al. reported a worse overall BS score and lower PA in doctors with shorter work experience, which was also confirmed by Makara-Studzinska et al. [43,55]. Furthermore, in a study by Głębocka et al. seniority was positively correlated with satisfaction from PA [8]. Reports of a higher incidence of BS in residents and physicians with shorter professional experience were also published on other populations. This relationship applies to both surgical and non-surgical specialties [73,74,75]. However, Dyrbye et al. conducted a survey in a large group of over 7000 doctors and found middle career (11–20 years in profession) to be particularly challenging time for doctors [76], also when analyzed by gender, specialty or setting. In the retrieved studies a very similar observation was made by Czekajska-Czehab et al. [36].

### 4.5. Working Time and BS in Physicians

Three of the selected studies indicated a relationship between the length of work or the number of jobs and the occurrence of BS in Polish doctors. The working hours along with the number of jobs, as well as shifts were assumed to be related to the risk of BS [36,45,55]. Ogińska-Bulik also emphasized that even a sense of work overload was associated with a more common occurrence of EE [37]. In addition, Makara-Studzinska et al. showed a protective effect of frequent use of annual leave [55]. Unfortunately, currently in Poland about 1 in 5 doctors reached retirement age [65], which will probably contribute to widening the existing staff shortages, a greater workload of active professional doctors and, as a result, often longer working hours.

### 4.6. Personality, Coping Strategies and Personal Resources and BS in Physicians

The articles contained in this systematic review identified a variety of other BS risk as well as protection factors. 

In an series of publications on the type of careers the authors identified three types of medical careers: “Committed—satisfied with career”, which are physicians committed to their work the most with the highest level of work stress; “Clever—satisfied with life”, the least committed to their work, but deriving the most benefit from it; and “Bright—competent”, which are the most competent, but with problems managing their lives. The “Committed” type proved to be at the highest risk of BS, whereas “Clever” at the lowest [46,47,48,49,50]. Other papers in our review have also identified individual character or personality traits as potentially significant factors. For example, Ogińska-Bulik et al. showed a greater severity of BS in healthcare professionals with type D personality [37]. On the other hand, Orzechowska et al. emphasized the protective importance of a strong internal locus of control [40], whereas Walocha et. al. of high level of empathy [44]. It may suggest that there are individuals who are a priori more susceptible to BS due to their personality. Brown et al. proposed specific personality traits that may particularly contribute to BS: neuroticism, agreeableness, and conscientiousness [77]. Pejušković et al. examined doctors for personality dimensions, stress coping strategies and the level of BS and noticed that harm avoidance led to a more common occurrence of BS, while self-directedness and cooperativeness were significantly more prevalent features in physicians with a lower level of BS [78]. 

From personal resources, the protective role against BS has been proven for, among others, satisfaction with social and family life, freedom, happiness, mature love, self-respect and a high level of support provided by superiors and co-workers [42,45,46,47,48,49,50].

### 4.7. Interventions to Prevent BS 

There is a large variety of possibilities of BS-focused interventions in doctors. In general, they may be classified in two main categories: interventions directed on the individuals and these targeting organization as a whole [79,80]. Physician-directed interventions may include cognitive behavioral therapy, training of communication skills, stress-coping strategies, creation support groups or improvement of opportunities for professional growth. Examples of organization-directed interventions can be given: rescheduling, reductions in the workload, greater institutional support, systematization of work and duties division. In the past few years, two important systematic reviews and metanalyses on this topic have been published [81,82]. The article by Panagioti et al. indicated that, unfortunately, intervention focused on decreasing BS in physicians were associated with rather small benefits. They underlined that organization-directed approaches might be slightly more effective than those geared to the individual. A review conducted by West et al., however, indicated that both individual-focused and organizational-focused approaches may be beneficial and suggested a combination of both tactics. Furthermore, in 2017 scientists from the Mayo Clinic have published their experiences with implementing organizational strategies therein and claimed that many simple and affordable interventions are effective, yet proper leadership and sustained attention seem to be the keys to success [83]. Currently, a clinical trial named Internet-Based Intervention for Occupational Stress Among Medical Professionals (Med-Stress) is carried out in Poland. It aims to examine the effectiveness of cognitive–behavioral therapyframed internet intervention for reduction of occupational stress, BS and depression (NCT03475290) [84]. Such affordable yet effective strategies might be the light at the end of the tunnel for doctors in Poland and abroad.

### 4.8. Advantages and Limitations of the Study

This review has several limitations that need to be mentioned. Above all, no quantitative analysis was performed due to the variety of tools used to assess the phenomenon of BS. Moreover, most commonly those studies did not allow dichotomous statement of burnout or the lack thereof, but rather indicated the existence and severity of some symptoms and it is therefore difficult to draw conclusions regarding the overall prevalence of this phenomenon. The included studies also did not cover doctors of all specialties and settings, so it is not entirely clear whether the conclusions drawn can be extrapolated to all doctors practicing in Poland. Another aspect relevant for interpretation limitation is the fact that the studies included in this review were mostly of medium or low reliability.

Nevertheless, to our knowledge no systematic review of the topic of BS in Polish doctors has been published to date. Therefore, this article is the most recent summary of available knowledge about BS in polish physicians in many aspects: prevalence, severity, its risk and protective factors. Moreover, it indicates the existing gaps in knowledge about this phenomenon, which we hope will become a trigger for further studies in this field. Despite the gaps in knowledge, based solely on the available research, it can be concluded with great certainty that the BS problem among Polish physicians is vivid; therefore, preventive and therapeutic actions should no longer be postponed. This is an important signal for both policy makers and physicians themselves.

## 5. Conclusions

BS is a multidimensional phenomenon that depends on various factors, such as personality traits, the nature of the work, management in the healthcare system, and many others. The results of individual studies are diverse, which makes it difficult to draw specific conclusions. However, the problem of burnout among Polish doctors is vivid and worth special attention from society, health policy leaders, and doctors themselves. High-quality research is essential for a better understanding of this topic.

## Figures and Tables

**Table 1:** Full electronic search strategy.

Database	Number of Results	Search Strategy
Pubmed	135	(“Physicians”[Mesh] OR doctor OR physician OR specialist OR healthcare professional OR health professional) AND (“Burnout, Professional”[Mesh] OR “Burnout, Psychological”[Mesh] OR burnout OR burned out) AND (“Poland”[Mesh] OR Poland OR Polish)
Scopus	92	(TITLE-ABS-KEY (doctor* OR physician* OR specialist* OR (healthcare AND professional*) OR (health AND professional*)) AND TITLE-ABS-KEY (burnout OR (burned AND out)) AND TITLE-ABS-KEY (Poland OR Polish))
CENTRAL	2	#1** “Physicians”[Mesh] #2 doctor* OR physician* OR specialist* OR (healthcare AND professional*) OR (health AND professional*) #3 “Burnout, Professional”[Mesh] #4 “Burnout, Psychological”[Mesh] #5 burnout OR (burned AND out) #6 “Poland”[Mesh] #7 Poland OR Polish #8 (#1 OR #2) AND (#3 OR #4 OR #5) AND (#6 OR #7)
Google Scholar (in English)	First 50*	(doctor* OR physician* OR specialist* OR (healthcare AND professional*) OR (health AND professional*)) AND (burnout OR (burned AND out) AND (Poland OR Polish))
Google Scholar (in Polish)	First 50**	(wypalenie OR (wypalenie AND zawodowe)) AND (lekarz* OR specjalist*) AND (Polska OR polsk* OR polsc*)

*Review of 50 first results out of 63,600 arranged in default order by database algorithms; **Review of 50 first results out of 429 arranged in default order by database algorithms. **Subsequent numbers correspond to successive lines entered in the CENTRAL search engine.

**Table 2:** Characteristics of the research articles included in the review.

Study	Final Number of Examined Physicians and Gender Distribution	Age*	Specialty	Seniority *
[36]	70 ♀ 66% ♂ 34%	Range 27–61	Radiology	12
[37]	51	40	Psychiatry	9
[38]	41 ♀ 56% ♂ 44%	38	Various	15
[39]	84 ♀ 48% ♂ 52%	=<40—28% 40–50—43% =>50—29%	Neurology—29% Cardiology—29% Orthopedics—24% Endocrinology—19%	LoD
[40]	55	Range 27–60	Internal medicine, surgery, pediatrics, social welfare home	Range 1–30
[41]	150	LoD	Full-time general practitioners or family medicine	LoD
[42]	82 ♀ 59% ♂ 41%	37	Surgery, orthopedics—44% Internal medicine, psychiatry, family medicine, oncology—56%	11
[43]	66 ♀ 71% ♂ 29%	48	Anesthesiology—52% Dermatology, ophthalmology—48%	1–13—53% 14–28—35% 29–43—12%
[44]	71 ♀ 35% ♂ 64%	Range 25–68	Primary care—41%; Non-surgical—32%; Surgical—27%	LoD
[45]	136 ♀ 52% ♂ 48%	49	Anesthesiology	23
Series: [46,47,48,49,50]	54 ♀ 69% ♂ 31%	29	Various	LoD
[8]	48	43	LoD	LoD
Series: [51] [52]	373 ♀ 58% ♂ 42%	42 in the whole study group	Anesthesiology	16
[53]	50	LoD	Various	LoD
[54]	25 ♀ 64% ♂ 36%	37	Emergency Medical Services	11
[55]	318 ♀ 66% ♂ 34%	47	42 different specialties, including: 18% of psychiatry, 16% of internal medicine, 13% of pediatrics	LoD

* Mean unless stated otherwise. Abbreviations: ♀—women; ♂—men; BS—burnout syndrome; DP—depersonalization; EE—emotional exhaustion; LoD—lack of data; MBI—Maslach Burnout Inventory; PA—personal accomplishment.

**Table 3:** Prevalence and the severity of burnout syndrome (BS).

Study	Main Burnout Assessment Tool	Level of Burnout Definition	Prevalence and Severity of BS in Physicians	Severity of BS Components in Physicians
[40]	MBI version not specified			High EE: 22% High DP: 62%
[41]	22-item MBI for Human Services Survey Polish translation	EE: low <13, average 14–26, high >27 DP: low <5, average 6–9, high >10 PA: high <33, average 34–39, low >40		High EE: 48% High DP: 34% Low PA: 30%
[42]	22-item MBI Polish adaptation by Pasikowski	Scores trichotomized with ½ of SD above and below the mean as cut points		High EE: 52% High DP: 35% Low PA: 34%
[8]	MBI version not specified			High EE: 33% High DP: 35% High PA: 32%
[37]	22-item MBI Polish adaptation by Pasikowski			For the nurses + physicians Mean: EE: 22, DP: 5, PA: 30
[38]	22-item MBI Polish adaptation by Pasikowski			Mean: ♀ EE: 20 ♂ EE: 21 ♀ DP: 8 ♂ DP: 9
[43]	22-item MBI version not specified	High overall stress ≥ 49	Mean overall result Anesthesiologists: 52 Other specialties: 41	Anesthesiologists: Mean EE: 27, DP: 9, PA: 16 Other specialties: Mean EE: 20, DP: 7, PA: 15
[44]	22-item MBI version not specified		Mean overall results: Surgical specialties: 58 Non-surgical: 59 Primary care: 53	Surgical: Mean EE: 20, DP: 8, PA: 29 Non-surgical: Mean EE: 22, DP: 9, PA: 28 Primary care: Mean EE: 22, DP: 10, PA: 29
[36]	66-item Burnout Scale by Studen and Okła	Stens scale: low <4, medium 4–6, high >6 overall result: low <3.34, medium 3.34–6.66, high >6.66	Low: 11% Medium: 51% High: 37% All symptoms at a high level: 16%	High level of: - PF: 53% - RE: 41% - LC: 40% - RC: 40% - SC: 36%
[39]	66-item Burnout Scale by Studen and Okła	Moderate 4–6 stens; Theoretical average for this scale -132	Most examined doctors do not experience significant burnout (mean 138)	Stens: - PF: 6 - RE: 6 - LC: 6 - RC: 5 - LC: 5
[53]	24-item Polish adaptation of Link Burnout Questionnaire		Mean: 60 (maximum 144)	
[55]	24-item Polish adaptation of Link Burnout Questionnaire	High ≥8 stens	Every second doctor showed high level of BS	High level of: - psychophysical exhaustion: 43% - commitment to relationship with patients: 44% - effectiveness in work: 47% - existential expectations: 48%
Series [51,52]	20-item Polish adaptation of Spanish Burnout Inventory	Critical level- values equal to or higher than 90th percentile	High or critical: 18%	The weakest possible rating of: - enthusiasm towards job: 20% - psychological exhaustion: 11% - indolence: 13% - feeling of guilt: 6%
[54]	36-item questionnaire regarding: attitude, stress, workload, contact with patient	Low risk <100 High risk >161	Mean: 131 Low risk: 18% High risk: 16%	

Abbreviations: BS—burnout syndrome; DP—depersonalization, EE—emotional exhaustion, LC—limited interpersonal contacts; MBI—Maslach Burnout Inventory; PA—personal accomplishment; PF—physical fatigue; RE—reduced effectiveness of action; RC—reduced emotional control; SC—loss of subject’s commitment.

**Table 4:** Risk and protective factors for BS.

Study	Factors Contributing to the More Frequent or Increased Incidence of BS	Protective Factors
[36]	- practicing 10–20 years - number of workplaces	- long work experience (≥20 years)
[37]	- work overload - negative affectivity - female (higher level of stress) - lack of rewards - physical burden - unpleasant work conditions - type D personality	
[38]	- sense of loss resources in last 12 months: hedonistic and vital, spiritual, family, material and political, power and prestige - cynicism	
[39]	- specialty (neurology)	- high score in Generalized Self-Efficacy Scale
[40]		- strong internal locus of control
[41]	- low job satisfaction - not having further qualification	
[42]	- high level of psychophysical demands and of requirements associated with responsibility for other people’s safety - female (lower self-perceived personal accomplishment)	- high level of intellectual demands, - individual cognitive control - high level of support provided by superiors and co-workers - self-enhancing humor style
[43]	- shorter professional experience	
[44]	- non-surgical specialty	- high level of empathy
[45]	- high workload (>60 hours a week)	- satisfaction with social life - good health
Series: [46,47,48,49,50]	2011: low level of the sense of comprehensibility in the 6th, high level of “trait” anxiety in the 4th, high level of depression in the 3rd, low need for social approval in the 3rd year of medical school 2012: - type of career “Committed—satisfied with career”—the most committed to their work with the highest level of work stress	2012: - type of career “Clever—satisfied with life”—the least committed to their work, but deriving the most benefit from it - well-being and life satisfaction 2014: - high sense of coherence at medical studies 2016: - taking actions and dealing directly with the problem at medical studies 2018: - family, freedom, happiness, mature love, self-respect, social recognition and wisdom
[8]	- negative assessment of healthcare, - poor relationships between healthcare professionals - positive assessment of conditions offered to in-patients - positive patients’ attitude toward physicians - negative patients’ attitude toward nurses	- seniority - positive general assessment of healthcare, - negative assessment of patients’ attitude toward physicians - proper relationship between healthcare professionals
[55]	- high level of perceived stress - large number of duties per month	- seniority - frequent use of annual leave

## Supplementary Materials

The following are available online at https://www.mdpi.com/1660-4601/16/24/5026/s1, Figure S1: PRISMA 2009 Flow Diagram, Table S1: Risk of bias assessment with the use of modified and adapted Newcastle-Ottawa Scale, Table S2. Principles of awarding points to individual publications.
