# Supplementary material for: Occupational Burnout Syndrome in Polish Physicians: A Systematic Review
Source: Int J Environ Res Public Health. 2019 Dec 10;16(24):5026. doi: 10.3390/ijerph16245026 (PMC6950131; doi:10.3390/ijerph16245026)
Supplement: Supplementary file 1 [file ijerph-16-05026-s001.pdf]

Supplementary Material

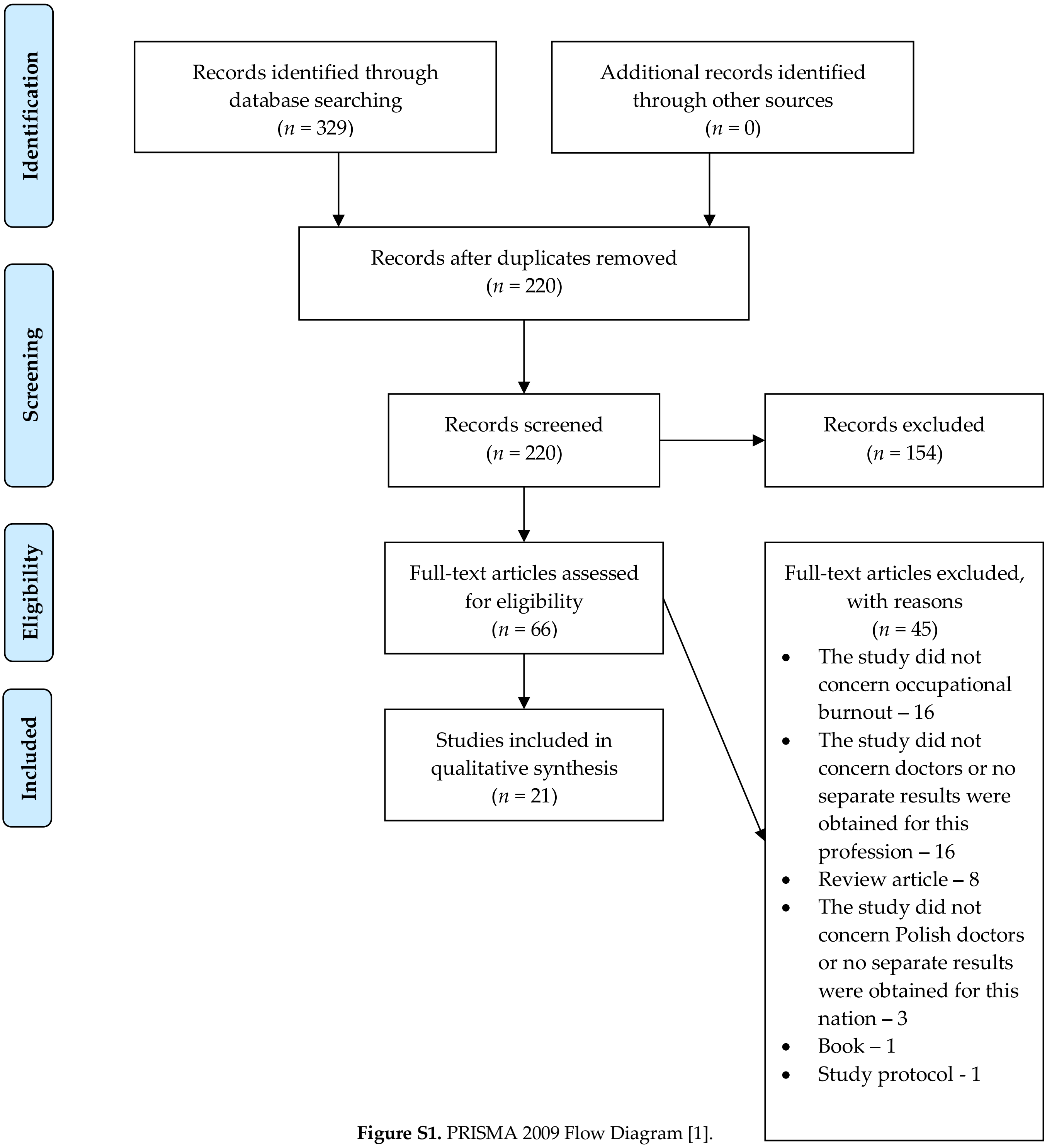

Figure S1. PRISMA 2009 Flow Diagram [1].

Table S1. Risk of bias assessment with the use of modified and adapted Newcastle-Ottawa Scale.

Study	Representativeness	Sample size	Non-Respondents	Ascertainment	Descriptive Statistics
[2]	0	0	0	1	0
[3]	0	0	0	1	1
[4]	1	0	0	1	1
[5]	1	0	0	1	1
[6]	1	0	0	1	0
[7]	1	0	1	1	1
[8]	1	0	0	1	1
[9]	1	0	0	1	0
[10]	1	0	0	1	0
[11]	0	0	1	1	1
Series: [12–16]	1	0	1	1	1
[17]	1	0	0	1	0
Series: [18,19]	0	1	1	1	1
[20]	1	0	0	1	0
[21]	0	0	1	1	1
[22]	1	1	1	1	1

Table S2. Principles of awarding points to individual publications.

Characteristics	0 points	1 point
Representativeness	Physicians of a single specialty and/or single institution	Physicians of multiple specialties and/or multiple institutions
Sample size	Number of Polish physicians participating in the study <200	Number of Polish physicians participating in the study ≥200
Non-respondents	No description of the response rate or no characteristics of the respondents and non-respondents	Exact response rate and/or comparability between respondent and non-respondent characteristics established
Ascertainment	Not enough details on the measurement tool/non-validated tool or a tool of uncertain validity	Well described measurement tool and/or validated tool
Descriptive statistics	Descriptive statistics of the population of Polish physicians were not reported, were incomplete, or did not include measures of dispersion	Descriptive statistics of the population of Polish physicians reported in details (e.g. age, sex, seniority) with measures of dispersion

Note: This is a modified version of the Newcastle-Ottawa Scale [23] used in a systematic review entitled 'Prevalence of Burnout Among Physicians. A Systematic Review' [24] adapted by the authors for the needs of this systematic review.

References

1. Moher, D.; Liberati, A.; Tetzlaff, J.; Altman, D.G.; The PRISMA Group (2009). Preferred Reporting Items for Systematic Reviews and Meta-Analyses: The PRISMA Statement. *PLoS Med* **2009**, *6*, e1000097, doi:10.1371/journal.pmed1000097.
2. Czekajska-Chehab, E.; Okla, W.; Drop, A.; Skrzypek, M. The professional burnout syndrome in radiologists. *Ann. Univ. Mariae Curie Sklodowska Med.* **2003**, *58*, 254–260.
3. Ogińska-Bulik, N. Occupational stress and its consequences in healthcare professionals: the role of type D personality. *Int. J. Occup. Med. Environ. Health* **2006**, *19*, 113–122.

4. Głębocka, A.; Lisowska, E. Professional burnout and stress among polish physicians explained by the Hobfoll resources theory. *J. Physiol. Pharmacol.* **2007**, *58*, 243–252.
5. Kulik, M.M. Cierpienie, które przerasta, czyli o wypaleniu lekarzy pracujących z ludźmi przewlekle chorymi. *Studia z psychologii w KUL. Wyd. KUL* **2008**, *15*, 81–112.
6. Orzechowska, A.; Talarowska, M.; Drozda, R.; Mirowska, D.; Florkowski, A.; Zboralski, K.; Gałecki, P. The burnout syndrome among doctors and nurses. *Polski Merkuriusz Lekarski* **2008**, *25*, 507–509.
7. Soler, J.K.; Yaman, H.; Esteva, M.; Dobbs, F.; Asenova, R.S.; Katić, M.; Ožvačić, Z.; Desgranges, J.P.; Moreau, A.; Lionis, C. Burnout in European family doctors: the EGPRN study. *Family practice* **2008**, *25*, 245–265.
8. Wojtyna, E.; Stawiarska, P. Humor styles and psychosocial working conditions in relation to occupational burnout among doctors. *Polish Psychological Bulletin* **2009**, *40*, 20–28.
9. Wypalenie zawodowe wśród personelu intensywnej opieki medycznej. Available online: https://repozytorium.ukw.edu.pl/handle/item/1030 (accessed on 10 December 2019).
10. Walocha, E.; Tomaszewski, K.A.; Wilczek-Ruzyczka, E.; Walocha, J. Empathy and burnout among physicians of different specialities. *Folia Med. Cracov.* **2013**, *53*, 35–42.
11. Gaszynska, E.; Stankiewicz-Rudnicki, M.; Szatko, F.; Wieczorek, A.; Gaszynski, T. Life satisfaction and work-related satisfaction among anesthesiologists in Poland. *ScientificWorldJournal* **2014**, *2014*, 601865, doi:10.1155/2014/601865.
12. Tartas, M.; Walkiewicz, M.; Majkowicz, M.; Budzinski, W. Psychological factors determining success in a medical career: A 10-year longitudinal study. *Med Teach* **2011**, *33*, e163–172, doi:10.3109/0142159x.2011.544795.
13. Walkiewicz, M.; Tartas, M.; Majkowicz, M.; Budzinski, W. Academic achievement, depression and anxiety during medical education predict the styles of success in a medical career: a 10-year longitudinal study. *Med Teach* **2012**, *34*, e611–619, doi:10.3109/0142159x.2012.687478.
14. Tartas, M.; Walkiewicz, M.; Budzinski, W.; Majkowicz, M.; Wojcikiewicz, K. The sense of coherence and styles of success in the medical career: A longitudinal study. *BMC Med Educ* **2014**, *14*, 254, doi:10.1186/s12909-014-0254-5.
15. Tartas, M.; Walkiewicz, M.; Budzinski, W.; Majkowicz, M.; Wojcikiewicz, K.; Zdun-Ryzewska, A. The coping strategies during medical education predict style of success in medical career: A 10-year longitudinal study. *BMC Med Educ* **2016**, *16*, 186, doi:10.1186/s12909-016-0706-1.
16. Budziński, W.; Walkiewicz, M.; Tartas, M. The system of values and styles of success in the medical career: A longitudinal study. *Int. J. Occup. Med. Environ. Health* **2018**, *31*, doi:10.13075/ijomeh.1896.01298.
17. Głębocka, A. The relationship between burnout syndrome among the medical staff and work conditions in the polish healthcare system. In *Advances in Experimental Medicine and Biology*, Spinger: Berlin, Germany **2017**; Vol. 968, pp 61–70.
18. Misiolek, A.; Gorczyca, P.; Misiolek, H.; Gierlotka, Z. The prevalence of burnout syndrome in Polish anaesthesiologists. *Anaesthesiol Intensive Ther.* **2014**, *46*, 155–161, doi:10.5603/ait.2014.0028.
19. Misiołek, A.; Gil-Monte, P.R.; Misiołek, H. Prevalence of burnout in Polish anesthesiologists and anesthetist nursing professionals: A comparative non-randomized cross-sectional study. *J. Health Psychol.* **2017**, *22*, 465–474, doi:10.1177/1359105315604377.
20. Grabowski, D.; Chudzicka-Czupała, A.; Chrupała-Pniak, M.; Rachwaniec-Szczecińska, Ż.; Stasiła-Sieradzka, M.; Wojciechowska, W. Work ethic, organizational commitment and burnout. *Med. Pr.* **2019**, *70*, 305–316, doi:10.13075/mp.5893.00800.
21. Leszczyński, P.; Panczyk, M.; Podgórski, M.; Owczarek, K.; Gałązkowski, R.; Mikos, M.; Charuta, A.; Zacharuk, T.; Gotlib, J. Determinants of occupational burnout among employees of the Emergency Medical Services in Poland. *Ann. Agric. Environ. Med.* **2019**, *26*, 114–119, doi:10.26444/aaem/94294.
22. Makara-Studzinska, M.; Zaluski, M.; Tylec, A.; Panasiuk, L. Do Polish doctors suffer from occupational burnout syndrome? An attempt to find an answer — Pilot study. *Ann. Agric. Environ. Med.* **2019**, *26*, 191–197, doi:10.26444/aaem/105392.
23. GA Wells, B.C., D.; Peterson, J.; Welch, V.; Losos, M.; Tugwell, P. The Newcastle-Ottawa Scale (NOS) for assessing the quality of nonrandomised studies in meta-analyses. http://www.ohri.ca/programs/clinical_epidemiology/oxford.asp (accessed on 28 October 2019).
24. Rotenstein, L.S.; Torre, M.; Ramos, M.A.; Rosales, R.C.; Guille, C.; Sen, S.; Mata, D.A. Prevalence of Burnout Among Physicians: A Systematic Review. *JAMA* **2018**, *320*, 1131–1150, doi:10.1001/jama.2018.12777.
